# Evaluating the necessity of prophylactic lateral neck dissection in medullary thyroid carcinoma based on preoperative calcitonin levels: a multicenter retrospective cohort study

**DOI:** 10.1097/JS9.0000000000003557

**Published:** 2025-10-03

**Authors:** Shin Jeong Pak, Douk Kwon, Byung-Chang Kim, Won Woong Kim, Tae Yon Sung, Ki-Wook Chung, Yu-Mi Lee, Jong Ju Jeong

**Affiliations:** a Department of Surgery, Hanyang University Hospital, Hanyang University College of Medicine, Seoul, Korea; b Department of Surgery, Asan Medical Center, University of Ulsan College of Medicine, Seoul, Korea; c Department of Surgery, Uijeongbu Eulji Medical Center, Eulji University, Uijeongbu, Korea; d Department of Surgery, Severance Hospital, Yonsei Cancer Center, Yonsei University College of Medicine, Seoul, Korea

**Keywords:** calcitonin, cohort study, medullary thyroid carcinoma, neck dissection, recurrence, survival rate

## Abstract

**Background:**

The need for prophylactic lateral neck dissection (LND) in medullary thyroid carcinoma (MTC) patients without clinically evident lateral lymph node (LN) metastasis remains controversial, particularly in those with elevated preoperative calcitonin levels. This study investigated the prognostic impact of prophylactic LND in MTC patients with preoperative calcitonin levels >200 pg/mL, but without clinically suspicious lateral LNs.

**Materials and Methods:**

This multicenter retrospective cohort study included 103 patients with MTC treated between January 1980 and December 2022 at two tertiary hospitals in Seoul, South Korea. Patients had preoperative calcitonin levels above 200 pg/mL and no clinical evidence of lateral LN metastasis. They were divided into two groups based on whether LND was performed, and their long-term oncological outcomes were compared.

**Results:**

The median follow-up duration was 90 months. The LND group had significantly larger tumors and higher preoperative calcitonin levels. Although the biochemical cure rate was higher in the LND group than in the No LND group (95.2% vs 82.5%), this difference was not statistically significant (*P* = 0.074). No significant differences were observed between the groups in biochemical recurrence, structural recurrence, overall survival, or disease-specific survival. Subgroup analyzes based on preoperative calcitonin levels showed comparable results.

**Conclusion:**

Prophylactic LND did not significantly impact long-term oncologic outcomes in MTC patients without clinically evident lateral LN metastasis, even among those with elevated preoperative calcitonin levels above 200 pg/mL. Although prophylactic LND was associated with higher biochemical cure rates, this did not translate into reduced recurrence rates or improved survival. Routine prophylactic LND may not be necessary, and the decision to perform it should be carefully individualized based on patient-specific factors.

**HIGHLIGHTS**

- Patients who underwent prophylactic lateral neck dissection had higher biochemical cure rates than those without dissection.
- However, no significant benefit in overall survival or disease-specific survival was observed with prophylactic lateral neck dissection.

## Introduction

Medullary thyroid carcinoma (MTC) is a distinct type of thyroid malignancy originating from parafollicular C cells. It is relatively rare, accounting for approximately 1–2% of all thyroid cancers. Despite its rarity, MTC often presents with regional lymph node (LN) metastasis, complicating its clinical management^[1,2]^. While MTC generally has a favorable prognosis, it is resistant to radioactive iodine therapy^[3,4]^. As a result, surgery remains the primary curative treatment option. Therefore, determining the appropriate extent of surgical intervention is critical for optimizing patient outcomes.

The standard treatment for MTC is typically total thyroidectomy, with LN dissection commonly recommended due to the high likelihood of LN metastasis^[1,4,5]^. However, the necessity of prophylactic LN dissection remains an ongoing debate. Although prophylactic central neck dissection (CND) is widely accepted as mandatory for all MTC patients^[5–7]^, the role of lateral neck dissection (LND) in patients without clinically suspicious lateral LNs remains controversial.

Decisions regarding prophylactic LND are often based on preoperative calcitonin levels. Calcitonin, a biomarker secreted by parafollicular C cells, indicates a higher risk of occult metastasis when elevated. The 2019 European Society for Medical Oncology (ESMO) guidelines recommend prophylactic ipsilateral LND for preoperative calcitonin levels between 50 and 200 pg/mL and suggest bilateral LND for calcitonin levels between 200 and 500 pg/mL^[8]^. In contrast, the 2015 American Thyroid Association (ATA) guidelines suggest considering prophylactic LND based on serum calcitonin levels but do not specify thresholds^[9]^. The National Comprehensive Cancer Network (NCCN) guidelines (version 1.2025) advise prophylactic ipsilateral LND for high-volume or gross MTC tumors, regardless of preoperative calcitonin levels^[10]^.

Despite these guidelines, recent studies have questioned the survival benefit of prophylactic LND in MTC patients with elevated preoperative calcitonin levels. Some experts advocate for a more aggressive approach, endorsing thorough prophylactic LND to enhance biochemical cure rates after surgery^[11–13]^. Conversely, other experts recommend a more conservative strategy due to potential complications and uncertain oncological benefits^[14–16]^. However, these studies often have limitations, including retrospective designs, single-center focus, and small sample sizes. Consequently, the necessity of prophylactic LND remains uncertain.

This study aims to investigate the prognostic impact of prophylactic LND in MTC patients with highly elevated preoperative calcitonin levels but without clinically suspicious lateral LN.

## Material and methods

### Study design and patient selection

**Figure 1.**
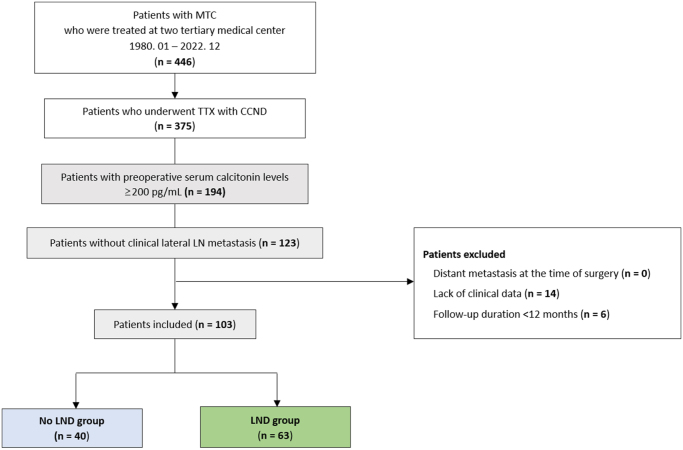
Flow chart of patient selection.

CCND, Central compartment node dissection; LN, Lymph node; LND, Lateral neck dissection; MTC, Medullary thyroid carcinoma; TTX, Total thyroidectomy

This multicenter retrospective cohort study was conducted at Asan Medical Center and Severance Hospital, two high-volume tertiary referral centers in Seoul, South Korea. Both institutions have longstanding experience in endocrine surgery, performing more than 2000 relevant surgical procedures annually. Between January 1980 and December 2022, 446 patients were treated for MTC at these hospitals (Fig. 1). Among them, 123 patients who underwent total thyroidectomy had preoperative calcitonin levels above 200 pg/mL and no clinical evidence of lateral LN metastasis. All patients underwent detailed preoperative evaluation, including neck ultrasound (US) with or without computed tomography (CT), to confirm the absence of suspicious lateral LN. Patients were excluded if they had distant metastasis before surgery, incomplete essential data (e.g., preoperative calcitonin levels, pathologic records), or a follow-up period of less than 12 months. Finally, 103 patients were included in this study. The patients were divided into two groups: those who did not undergo prophylactic LND (No LND group) and those who did (LND group). An analysis was conducted to evaluate the effect of prophylactic LND on MTC outcomes.

Data from patients’ medical records included demographic and clinical characteristics, such as the age at diagnosis, sex, preoperative serum calcitonin level, carcinoembryonic antigen (CEA) level, and duration of follow-up. Histopathologic findings including tumor size, microscopic/gross extrathyroidal extension (ETE), LN metastasis, and extra-nodal extension. Operation records and follow-up outcomes were also collected, including laboratory data (serum calcitonin and CEA levels), imaging studies, and oncologic outcomes. All data were reviewed retrospectively by three independent reviewers.

This study was approved by the institutional review board of both hospitals (No. 2023-87 0428 and No. 4-2024-1398). The need for informed consent was waived due to the study’s retrospective nature. The study was registered with the Research Registry (researchregistry11271), in accordance with the 2013 Declaration of Helsinki by the World Medical Association. The work has been reported in line with the strengthening the reporting of cohort, cross-sectional, and case-control studies in surgery (STROCSS) criteria^[17]^.

### Surgical technique and postoperative follow-up protocol

LND was performed using the standard compartment-oriented technique of modified radical neck dissection (MRND) to address potential LN metastasis, including levels 2, 3, 4, and occasionally 5. All surgeries were performed by experienced endocrine surgeons, each of whom performs over 200 thyroidectomies annually. The decision to perform LND was based on imaging findings (e.g., tumor size, characteristics, and the presence of central LN metastasis), preoperative calcitonin levels, and patient preferences. In cases where LND was performed, the surgeon decided whether to perform ipsilateral or bilateral LND after thorough discussions with the patients.

Postoperative evaluations included physical examination, serum calcitonin level, serum CEA level, and thyroid function test (TFT) at 3–6 months postoperatively. Subsequent follow-ups included assessments of the serum calcitonin level, CEA level, TFT, and US at 6-month to 1-year intervals. If the serum calcitonin level increased to 150 pg/mL or higher, or if recurrence was suspected, additional imaging studies such as chest CT, abdomen-pelvic CT, bone scans, and positron emission tomography-computed tomography (PET-CT) were conducted.

Serum calcitonin levels were measured by immunoradiometric assay human calcitonin (IRMA-hCT; CisBio International, Codolet, France), with a functional sensitivity of 1.2 mol/L, detection limit of 0.4 pmol/L, and reference upper limit of 2.9 pmol/L. The intra-assay coefficient of variation of the serum calcitonin assay was 1.2–6.7%, and the inter-assay coefficient of variation was 4.3–5.2%.

### Study endpoint

The primary endpoints were overall survival (OS) and disease-specific survival (DSS). Key secondary endpoints included a biochemical cure, biochemical recurrence, and structural recurrence. A biochemical cure was defined as normalizing calcitonin levels to 10 pg/mL or lower at 6–12 months postoperatively. Biochemical recurrence was defined as an increase in postoperative calcitonin levels above the upper normal limits in patients who had previously achieved a biochemical cure during follow-ups, or as more than a twofold increase compared to the lowest postoperative calcitonin level. Structural recurrence was defined as recurrence detected by imaging studies such as thyroid US, CT, bone scans, or PET-CT.

### Statistical analysis

Continuous variables were presented as medians with interquartile ranges (IQRs), whereas categorical variables were expressed as numbers with percentages (%). The Chi-squared test was used to compare the distribution of categorical variables (e.g., sex and gross ETE). In contrast, the Mann–Whitney nonparametric test was used for continuous variables. Bivariate analyses for continuous variables were performed using the Student’s *t*-test or Wilcoxon rank-sum test, depending on distribution, and the Chi-squared test was used for categorical variables. OS, DSS, structural recurrence-free survival, and biochemical recurrence-free survival were estimated using the Kaplan–Meier method and compared across subgroups using log-rank tests. Univariate and multivariate logistic regression models evaluated risk factors associated with outcomes. The results were presented as hazard ratios (HRs) and 95% confidence intervals (CIs). All significance tests were two-sided, and *P*-values less than 0.05 were considered statistically significant. Statistical analyses were performed using IBM SPSS Statistics for Windows, version 27.0 (IBM Corp., Armonk, NY, USA), R version 4.3.3, and R libraries (R Foundation for Statistical Computing, Vienna, Austria; www.R-project.org).

## Results

### Patient demographics and clinical characteristics

The demographics and clinical characteristics of the study patients are summarized in Table 1. A biochemical cure was achieved in 90.3% of the patients after surgery. The median follow-up period was 90 months (range: 13–311 months). During follow-up, 17 patients (16.5%) experienced biochemical recurrence, and seven (6.8%) had structural recurrence. There were seven deaths in total (6.8%), with one death (1.0%) attributed to MTC.

**Table 1:** Clinical characteristics and long-term outcomes (total patients)

	No LND group *N* = 40^a^	LND group *N* = 63^a^	Total *N* = 103^a^	*P* value
Age, years	55.5 (40.5–64.5)	51.0 (40.0–60.0)	54.0 (40.5–61.0)	0.359
Sex, female	26 (65.0)	44 (69.8)	70 (68.0)	0.767
Follow-up duration, months	78.0 (48.5–124.5)	122.0 (48.0–183.0)	90.0 (48.0–163.5)	0.128
Primary tumor				
Tumor size, cm	1.7 (1.2–2.2)	2.5 (1.8–3.2)	2.0 (1.5–3.0)	0.001
Gross ETE	3 (7.5)	4 (6.3)	7 (6.8)	>0.999
Clinical LNM				
Central	3 (7.5)	6 (9.5)	9 (8.7)	>0.999
Pathologic LNM				
Central	8 (20.0)	20 (31.7)	28 (27.2)	0.281
Ipsilateral lateral	N/A	18 (28.6)	18 (17.5)	-
Contralateral lateral	N/A	1 (1.6)	1 (1.0)	-
Extra-nodal extension	6 (15.0)	10 (16.1)	16 (15.7)	>0.999
Preop calcitonin, pg/mL	489.0 (276.0–872.5)	705.2 (406.7–1,490.0)	582.0 (314.5–1,232.5)	0.013
Preop calcitonin group				0.140
200 ≤, < 500 pg/mL	20 (50.0)	21 (33.3)	41 (39.8)	
≥500 pg/mL	20 (50.0)	42 (66.7)	62 (60.2)	
Elevated preop CEA	28/32 (87.5)	37/44 (84.1)	65/76 (85.5)	0.931
Postop calcitonin, pg/mL				
POD 6 months	1.5 (1.5–5.0)	1.5 (1.5–3.2)	1.5 (1.5–4.0)	0.992
POD 1 year	1.5 (1.1–5.6)	1.5 (1.5–3.0)	1.5 (1.5–3.8)	0.673
Elevated postop CEA				
POD 6 months	1/30 (3.4)	3/52 (5.8)	4 (4.9)	>0.999
POD 1 year	2/37 (5.4)	3/55 (5.5)	5 (5.4)	>0.999
Biochemical cure	33 (82.5)	60 (95.2)	93 (90.3)	0.074
Biochemical recurrence	9 (22.5)	8 (12.7)	17 (16.5)	0.301
Structural recurrence	3 (7.5)	4 (6.3)	7 (6.8)	>0.999
Disease-specific mortality	0 (0.0)	1 (1.6)	1 (1.0)	>0.999
Overall mortality	2 (5.0)	5 (7.9)	7 (6.8)	0.861

CEA, carcinoembryonic antigen; ETE, Extrathyroidal extension; LND, Lateral neck dissection; LNM, Lymph node metastasis; POD, postoperative day.

^a^ *n* (%); Median (IQR).

The 103 patients were divided into 40 in the No LND group and 63 in the LND group. There were no significant differences in age or sex distribution between the two groups. However, the LND group had significantly larger tumor sizes (*P* = 0.001) and higher preoperative calcitonin levels (*P* = 0.013). Postoperative calcitonin and CEA levels were similar between the groups. Although the LND group had a higher biochemical cure rate (95.2% vs 82.5%), this difference was not statistically significant (*P* = 0.074). Both biochemical and structural recurrence rates were similar between the two groups, and there were no significant differences in OS and DSS.

### Stratified analysis by preoperative calcitonin levels

Table 2 presents the characteristics of the No LND and LND groups, stratified by preoperative calcitonin levels. For patients with preoperative calcitonin levels between 200 and 500 pg/mL, no significant differences were observed between the No LND and LND groups. The biochemical cure rate was 90.0% in the No LND group and 100% in the LND group (*P* = 0.447), whereas biochemical recurrence rates were similar between the two groups (*P* = 0.612). Structural recurrence and overall mortality were also comparable between the groups.

**Table 2:** Comparison of baseline characteristics and long-term outcomes between preoperative calcitonin levels 200–499 pg/mL and ≥500 pg/mL

	Preop calcitonin 200–499 mL			Preop calcitonin ≥500 pg/mL		
	No LND group *N* = 20^a^	LND group *N* = 21^a^	*P* value	No LND group *N* = 20^a^	LND group *N* = 42^a^	*P* value
Age, years	59.5 (54.0–66.0)	57.0 (53.0–61.0)	0.2900	48.5 (36.0–60.5)	47.5 (37.0–60.0)	0.988
Sex, female	13 (65.0)	13 (61.9)	>0.999	13 (65.0)	31 (73.8)	0.678
Primary tumor						
Tumor size, cm	1.6 (1.1–2.0)	1.4 (1.2–2.1)	0.927	1.9 (1.4–2.2)	3.0 (2.0–3.5)	<0.001
Gross ETE	2 (10.0)	2 (9.5)	>0.999	1 (5.0)	2 (4.8)	>0.999
Preop calcitonin, pg/mL	276.0 (216.5–344.0)	303.4 (244.0–405.0)	0.361	872.5 (585.0–1,276.5)	1,225.0 (705.2–2,320.0)	0.070
Elevated preop CEA	10/14 (71.4)	10/14 (71.4)	>0.999	18/18 (100.0)	27/30 (90.0)	0.441
Postop calcitonin, pg/mL						
POD 6 months	1.5 (1.5–1.6)	1.5 (1.5–1.5)	0.455	3.4 (1.5–6.8)	1.5 (1.5–4.2)	0.328
POD 1 year	1.5 (1.5–2.9)	1.5 (1.5–1.9)	0.872	2.4 (1.5–13.2)	1.6 (1.5–3.8)	0.942
Elevated postop CEA						
POD 6 months	0/16 (0.0)	0/17 (0.0)	-	1/16 (6.2)	3/37 (8.1)	>0.999
POD 1 year	1/17 (5.9)	1/17 (5.9)	>0.999	1/20 (5.0)	2/38 (5.3)	>0.999
Biochemical cure	18 (90.0)	21 (100.0)	0.447	15 (75.0)	39 (92.9)	0.120
Biochemical recurrence	3 (15.0)	2 (9.5)	0.612	5 (25.0)	6 (14.3)	0.499
Structural recurrence	1 (5.0)	0 (0.0)	0.980	2 (10.0)	4 (9.5)	>0.999
Disease-specific mortality	0 (0.0)	0 (0.0)	-	0 (0.0)	1 (2.4)	>0.999
Overall mortality	1 (5.0)	1 (4.8)	>0.999	1 (5.0)	4 (9.5)	0.910

CEA, carcinoembryonic antigen; ETE, Extrathyroidal extension; LND, Lateral neck dissection; POD, postoperative day.

^a^ *n* (%); Median (IQR)

For patients with calcitonin levels of 500 pg/mL or higher, the LND group had significantly larger tumors compared to the No LND group (*P* < 0.001). Although preoperative calcitonin levels tended to be higher in the LND group compared to the No LND group, the difference was not statistically significant (*P* = 0.070). The biochemical cure rate was higher in the LND group, but this difference was not statistically significant (*P* = 0.120). Biochemical recurrence rates (*P* = 0.499), structural recurrence rates (*P* > 0.999), and overall mortality (*P* = 0.910) were also similar between the two groups.

### Long-term oncologic outcomes

Table 3 outlines the characteristics and outcomes of patients who experienced structural recurrence during follow-ups. Seven patients experienced structural recurrence: three in the No LND group and four in the LND group. In both groups, recurrence was observed in patients with high preoperative calcitonin levels. None of the patients in the No LND group achieved a biochemical cure. In the LND group, two patients achieved a biochemical cure, while the others did not. Despite recurrence, most patients remained alive, with one death in the LND group due to multiple distant metastases. This patient was the only one in our study cohort to die from disease-related causes.

**Table 3:** Structural recurrence during follow-up

Group	Sex	Age	Tumor size (cm)	pT	pN	Calcitonin (pg/mL)			Recurrence	Time to recurrence (years)	Last status
						Preop	Postop 6 months	Biochemical cure			
No LND	F	33	2.0	1	N/A	278	189.0	No	Ipsilateral level 2 LN	3.1	Alive
									Liver metastasis	4.7	
	F	35	2.2	2	N/A	877.0	19.1	No	Ipsilateral level 2, 3, 4, 5, 6 LN	10.1	Alive
	F	60	0.8	1	N/A	1008	909.0	No	Ipsilateral level 2, 4 LN	1.8	Alive
LND	F	36	2.0	1	1b	1105.0	1.5	Yes	Operative bed	17.5	Alive
	F	44	2.0	1	1b	1244.0	5.1	Yes	Ipsilateral level 2, 3, 4, 5 LN	12.0	Alive
	F	30	3.5	2	1a	3720.0	205.0	No	Liver metastasis	8.6	Alive
	M	56	3.0	3b	1b	>6700	1926.0	No	Lung, bone metastasis	1.0	Dead

LN, Lymph node; LND, Lateral neck dissection; N/A, not available.

**Figure 2.**
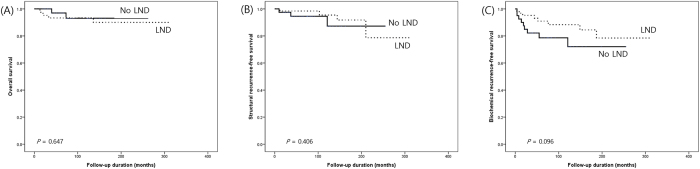
Long-term outcomes in medullary thyroid carcinoma patients based on lateral neck dissection status (A) Overall survival, (B) structural recurrence, and (C) biochemical recurrence.

Figure 2 shows the 10-year survival rates for the No LND and LND groups across OS, structural recurrence-free survival, and biochemical recurrence-free survival. For OS, the No LND group had a 10-year survival rate of 93.1%, while the LND group had a similar rate of 93.3% (Fig. 2(A), *P* = 0.647). Structural recurrence-free survival rates were high in both groups, with 10-year survival rates of 94.5% in the No LND group and 95.4% in the LND group (Fig. 2(B), *P* = 0.406). For biochemical recurrence-free survival, the 10-year rates were 78.6% and 88.3% in the No LND and LND groups, respectively. Although the No LND group showed a slightly lower biochemical recurrence-free survival rate in the early period, this difference was not statistically significant over the long term (Fig. 2(C), *P* = 0.096).

LND, Lateral neck dissection

**Figure 3.**
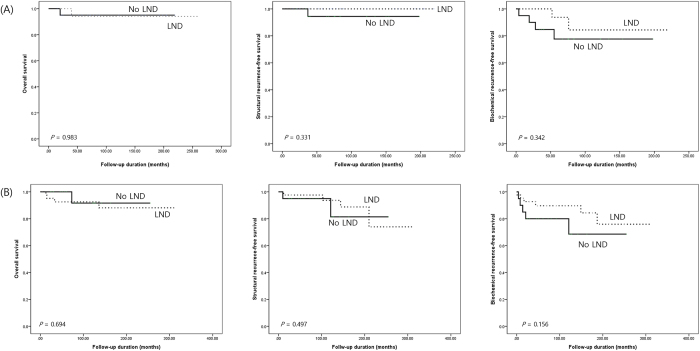
Subgroup survival analysis by preoperative calcitonin levels (A) Preop calcitonin 200–499 mL, (B) Preop calcitonin ≥500 pg/mL.

There were no statistically significant differences in OS, structural recurrence-free survival, and biochemical recurrence-free survival between the No LND and LND groups, even when stratified by calcitonin levels (500 pg/mL cutoff). Biochemical recurrence-free survival showed a trend favoring the LND group for calcitonin levels below 500 pg/mL. However, this did not reach statistical significance (Fig. 3).

LND, Lateral neck dissection

The Cox regression analysis identified significant risk factors associated with structural recurrence (Table 4). Tumor sizes ≥2 cm significantly predicted structural recurrence (*P* = 0.032). At the same time, the extra-nodal extension was significant for biochemical recurrence (*P* = 0.001). Biochemical failure was a strong predictor of both biochemical recurrence (*P* < 0.001) and structural recurrence (*P* < 0.001). However, the no LND group was not significantly related to either biochemical or structural recurrences. No significant risk factors for OS were identified, and DSS analysis was not feasible because of the very low number of events (only one case).

**Table 4:** Analysis of risk factors for structural recurrence using Cox regression

Variable	Biochemical recurrence				Structural recurrence			
	Univariate		Multivariate		Univariate		Multivariate	
	HR (95% CI)	*P* value	HR (95% CI)	*P* value	HR (95% CI)	*P* value	HR (95% CI)	*P* value
Sex, male	0.86 (0.25–2.58)	0.800			0.33 (0.02–2.07)	0.319		
Age, ≤40 years	1.80 (0.56–5.38)	0.301			4.48 (0.92–24.2)	0.061		
Tumor size, ≥2 cm	0.98 (0.34–2.93)	0.971			4.47 (0.73–86.2)	0.173	20.60 (1.30–327.74)	0.032
Gross extrathyroidal extension	2.16 (0.29–11.10)	0.383			2.50 (0.12–18.4)	0.429		
Preop calcitonin levels ≥500 pg/mL	1.26 (0.44–3.94)	0.678			4.29 (0.69–82.6)	0.186		
Clinical central LNM	1.50 (0.21–6.98)	0.631			1.83 (0.09–12.8)	0.595		
Extra-nodal extension	12.5 (3.77–45.00)	<0.001	5.96 (2.00–17.73)	0.001	4.73 (0.85–24.0)	0.058		
No LND group	2.00 (0.70–5.83)	0.197			1.20 (0.23–5.72)	0.821		
Biochemical failure	95.6 (15.3–1885)	<0.001	17.39 (5.99–50.50)	<0.001	45.5 (7.86–383)	<0.001	81.92 (8.64–776.48)	<0.001

CI, Confidence Interval; HR, Hazard Ratio; LND, Lateral neck dissection; LNM, Lymph node metastasis.

## Discussion

This multicenter retrospective cohort study found that prophylactic LND did not significantly impact long-term oncological outcomes in MTC patients with preoperative calcitonin levels above 200 pg/mL and no clinical evidence of lateral LN metastasis. Although prophylactic LND facilitated a biochemical cure, especially in patients with preoperative calcitonin levels exceeding 500 pg/mL, it did not reduce recurrence rates or enhance long-term survival.

Determining the appropriate surgical extent of LND in patients with MTC is critical for optimizing outcomes. A previous study by Machens and Dralle *et al* reported that lateral LN metastases were often detected when serum calcitonin levels exceeded 200 pg/mL^[18]^. Additionally, higher calcitonin levels were associated with lower biochemical cure rates and increased incidences of upper mediastinal LN metastasis and distant metastasis^[3,19,20]^. Based on these findings, various guidelines have recommended prophylactic LND for patients with elevated calcitonin levels^[8–10,21]^.

However, more recent studies have challenged this approach. Spanheimer *et al* observed that prophylactic ipsilateral LND did not improve survival rates in MTC patients with preoperative calcitonin levels above 200 pg/mL and found no clinical or radiologic LN metastasis^[14]^. Similarly, Ansley *et al* found no significant difference in disease-free survival between patients who underwent central node dissection alone and those who underwent bilateral LND in a high calcitonin group^[15]^.

Consistent with recent studies, our results demonstrated that prophylactic LND did not significantly impact long-term oncologic outcomes, even in patients with high preoperative calcitonin levels. Structural recurrence occurred in 3 out of 40 patients (7.5%) in the No LND group and 4 out of 63 (6.3%) in the LND group during the follow-up period. Although the biochemical recurrence rate was higher in the No LND group, the difference was not statistically significant. Disease-specific mortality was extremely low, occurring in only one patient (1.6%). Furthermore, subgroup analysis based on a preoperative calcitonin threshold of 500 pg/mL revealed no significant differences between the No LND and LND groups.

These findings suggest that routine prophylactic LND may not be necessary in MTC patients without clinically evident lateral LN metastasis, even in those with elevated preoperative calcitonin levels. LND is an extensive surgical procedure associated with potential morbidities, including cosmetic issues, bleeding or hematoma, chyle leakage, nerve injury, vessel injury, and adhesion. Such complications, which were not systematically captured in our dataset, can significantly impact patients’ quality of life and represent an important area for future prospective research. Therefore, a balanced consideration of the potential benefits and risks of LND is essential. Individualized surgical approaches based on patient-specific factors and preferences may optimize outcomes while minimizing unnecessary morbidities.

Previous studies have highlighted the role of prophylactic LND in improving biochemical cure rates for MTC. A biochemical cure is considered as an important marker of surgical success and disease control^[4,11–13,18,22]^. Machens *et al* reported that the extent of LND was closely associated with achieving a biochemical cure, particularly in patients with elevated preoperative calcitonin levels^[18]^. Removing microscopic lateral LN metastasis through prophylactic LND reduces residual disease burden, thereby increasing the likelihood of achieving a biochemical cure. Our study also demonstrated that the LND group had a higher biochemical cure rate compared to the No LND group (95.2% vs 82.5%). Although it did not reach statistical significance, this difference was more pronounced in patients with preoperative calcitonin levels exceeding 500 pg/mL. Furthermore, the multivariable analysis identified failure to achieve a biochemical cure as a significant risk factor for both structural recurrence [hazard ratio (HR) = 81.92, *P* <0.001] and biochemical recurrence (HR = 17.39, *P* < 0.001).

Nevertheless, a biochemical cure did not necessarily prevent structural recurrence in our study. Two patients who experienced recurrence in the LND group had achieved biochemical cure. This indicated that biochemical normalization alone might not be a reliable predictor of long-term disease control. Given that prophylactic LND improved biochemical cure rates but did not significantly impact recurrence or survival, its routine use should be carefully reconsidered. The decision should be individualized, balancing potential oncologic benefits against surgical morbidity.

This study has several limitations. First, despite being a multicenter study from two tertiary hospitals, the small sample size due to the rarity of MTC limits the generalizability of our results. Second, the retrospective nature of our study may have introduced data limitations, including incomplete evaluation of important prognostic factors such as RET gene mutation status, Ki-67 proliferation index, tumor necrosis, and mitotic count, which are recommended in the recent WHO classification and may influence the risk of lateral LN metastasis^[23]^. Additionally, some patients may not have undergone sufficient preoperative metastasis evaluation (e.g., CT, PET-CT scans), potentially affecting accuracy. Third, despite long-term follow-up, the low event rates (death and recurrence) made it challenging to identify significant risk factors, affecting the statistical power. Fourth, surgical complications and quality-of-life outcomes were not systematically recorded, which limited our ability to assess the functional and patient-centered impact of prophylactic LND. Lastly, selection bias may have occurred due to the retrospective nature of this study. Prophylactic LND may have been more likely chosen for patients with advanced MTC based on the tumor size or US findings, potentially impacting our results. Therefore, further studies with larger sample sizes and prospective or randomized controlled trials are needed to confirm our findings.

Nonetheless, this study is meaningful because it draws upon long-term data from two major tertiary hospitals in Korea to evaluate whether prophylactic LND is necessary in MTC patients with high calcitonin levels but no radiologic evidence of lateral LN metastasis. Our findings may contribute to establishing guidelines regarding the extent of surgery for MTC patients and offers real-world evidence supporting individualized surgical decision-making in this patient group.

In conclusion, our multicenter retrospective cohort study indicated that prophylactic LND does not significantly impact long-term oncological outcomes in MTC patients without clinically evident lateral LN metastasis, even among those with elevated preoperative calcitonin levels exceeding 200 pg/mL. Although prophylactic LND was associated with higher biochemical cure rates – particularly in patients with calcitonin levels exceeding 500 pg/mL– this did not translate into reduced recurrence rates or improved overall survival. Given the potential morbidities of LND and the absence of demonstrated survival benefit, our findings reinforce current guideline recommendations against routine prophylactic LND in the absence of clinical or radiologic evidence of metastasis. Therefore, routine prophylactic LND may not be necessary for all MTC patients; and the decision should be carefully individualized, incorporating patient-specific risk factors, molecular and pathological profiles when available, and patient preferences, to balance potential benefits against operative risks.

## Data Availability

Due to the inclusion of patients’ personal and medical information, the data used in this study cannot be shared to ensure the protection of patient confidentiality.

## Research registration unique identifying number (UIN)

Name of the registry: The Research Registry; Unique identifying number or registration ID: researchregistry11271; Hyperlink to the registration: https://www.researchregistry.com/browse-the-registry#home/.
